# Obturator Bypass with Bovine Carotid Artery Graft: A Novel Twist to a Traditional Technique

**DOI:** 10.1155/2019/2653058

**Published:** 2019-02-19

**Authors:** Sarah Brown, Christopher Esper, Jon Henwood

**Affiliations:** Department of Surgery, University of Pittsburgh Medical Center, Pittsburgh, PA, USA

## Abstract

When managing an infected groin, though rarely performed, a transobturator bypass remains an important alternative in the armament of a vascular surgeon. Autologous vein and synthetic PTFE are known conduit options for obturator bypasses, although the advantage of utilizing an autologous biological conduit when dealing with infection may not be an option for every patient. On literature review, bovine carotid grafts have been used in infrainguinal revascularizations with comparable results to autologous vein; however, no cases can be found for its use in obturator bypass thus far.

## 1. Introduction

Infection of bypass grafts occur most commonly in the groin secondary to anatomic location, disruption of lymphatics during dissection, and superficial location of the graft itself. Graft infection complications can include bacteremia, sepsis, bleeding, and acute limb ischemia. Complete graft explantation is required to prevent reinfection, and primary revascularization should be considered first-line treatment due to high rates of lower extremity amputation after ligation [1]. The obturator bypass has advantages in cases of groin infection as the deep route of an obturator bypass circumvents infected fields as well as a shorter graft length. In literature review, extraperitoneal or transperitoneal approaches as well as varying conduit type with either autologous vein or synthetic grafts such as PTFE are encountered; however, we present a case for an obturator bypass with bovine carotid conduit and explantation of infected femorofemoral bypass graft [2, 3].

## 2. Case Report

**Figure 1:**
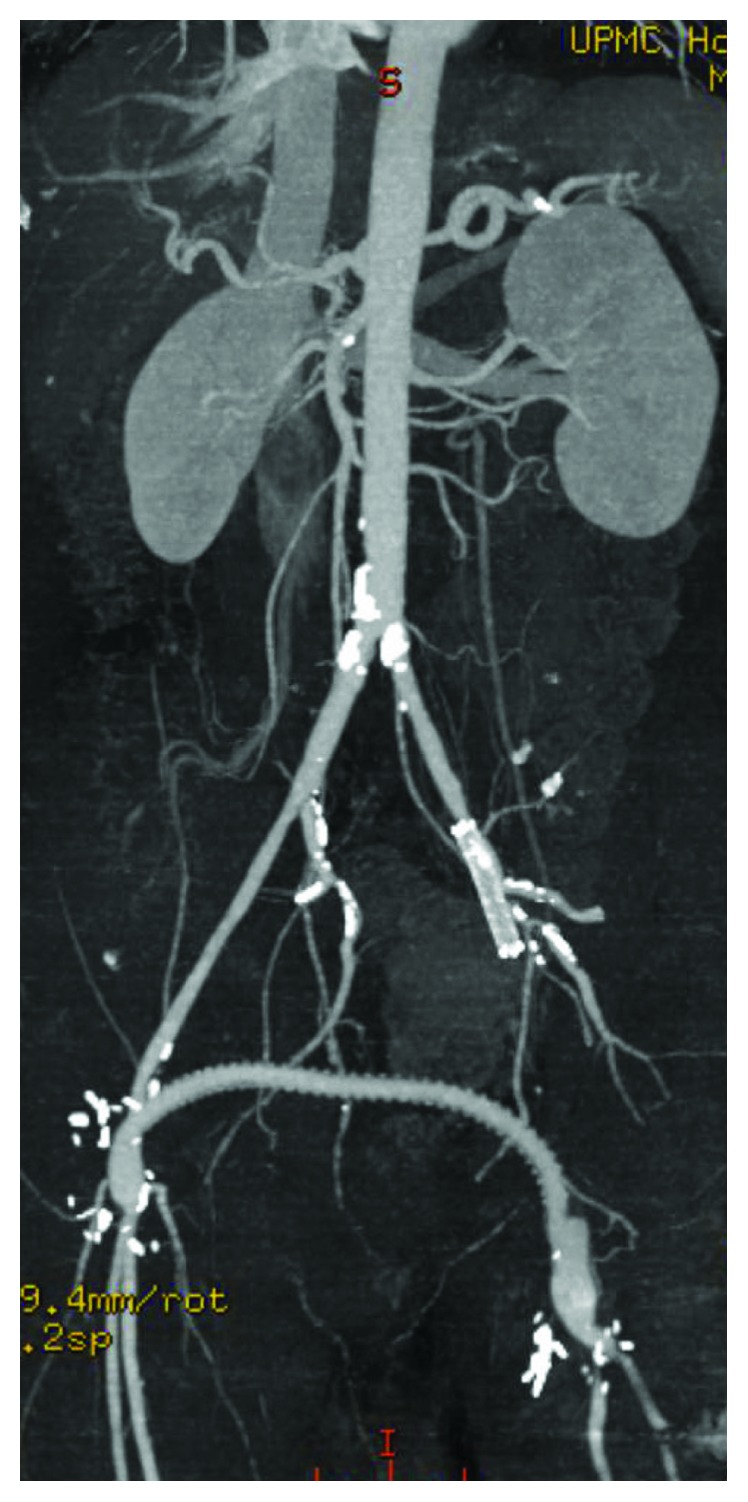
Previous right to left femorofemoral bypass with left external iliac stent occlusion.

**Figure 2:**
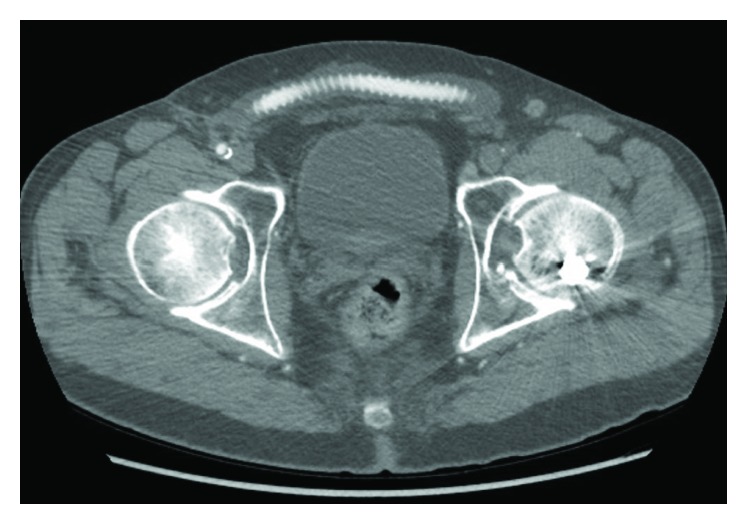
Fluid surrounding the femorofemoral bypass graft.

**Figure 3:**
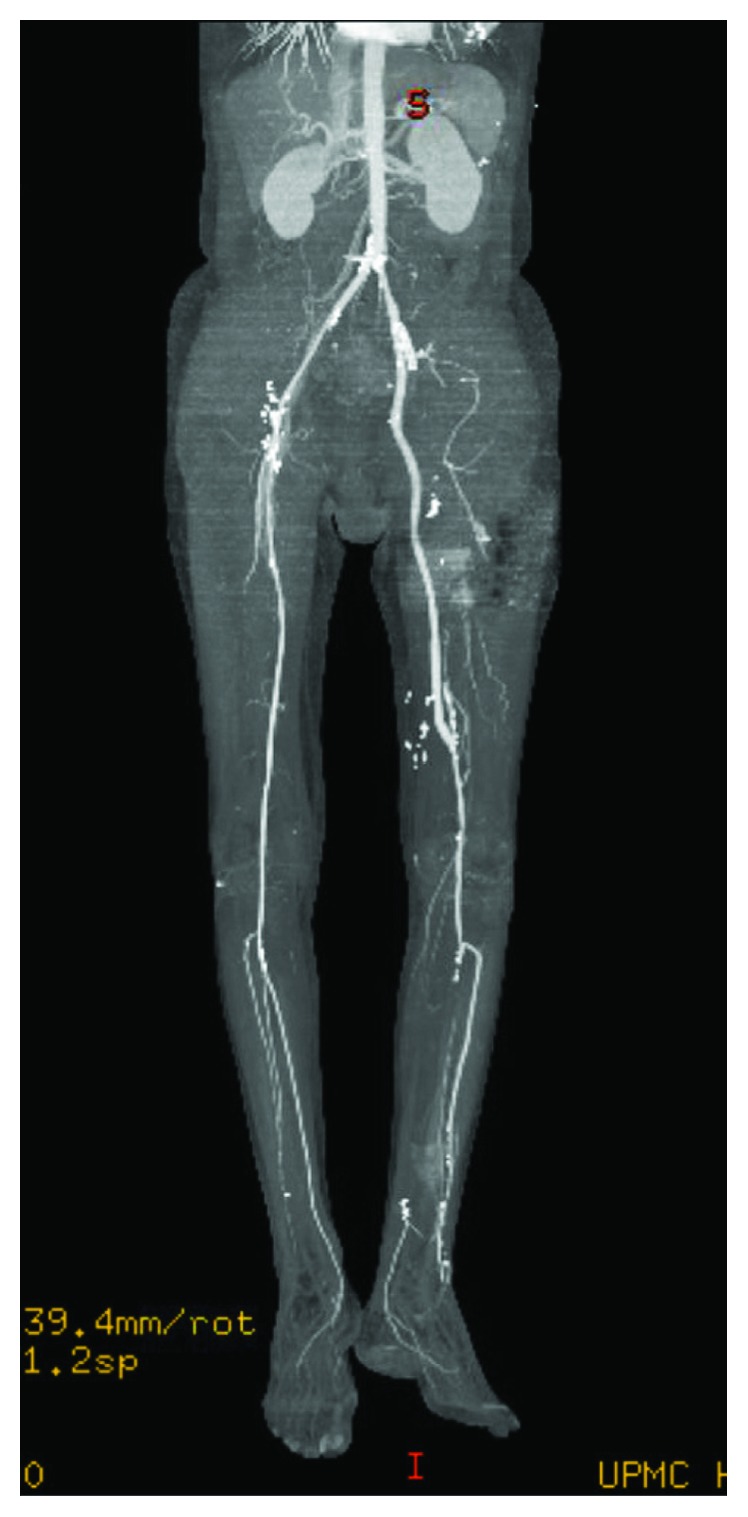
Postoperative CT angiogram of obturator bypass.

**Figure 4:**
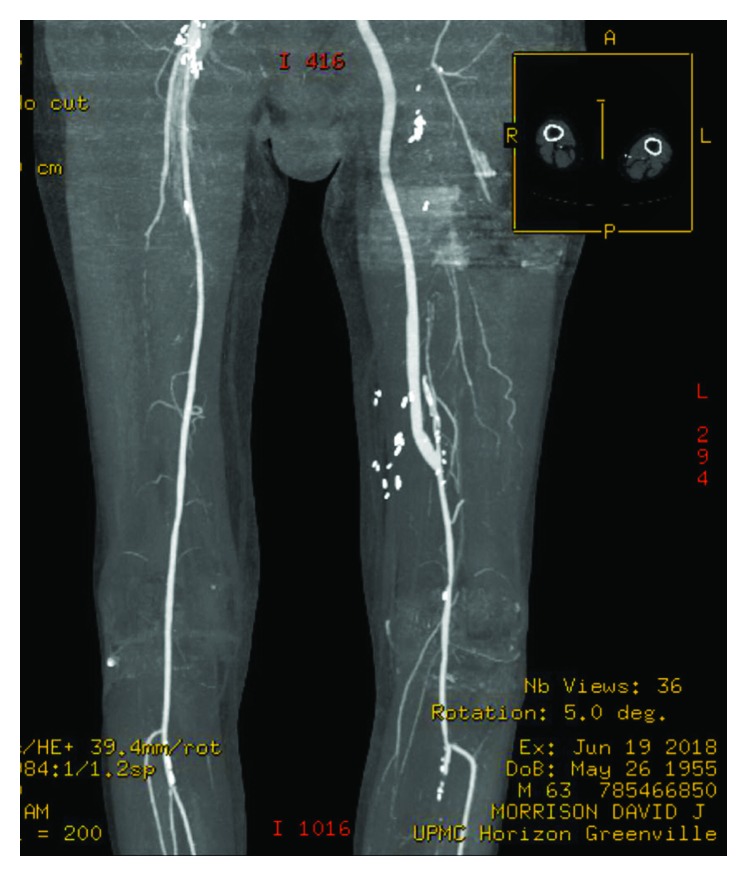
Postoperative CT angiogram of obturator bypass demonstrating distal anastomosis and runoff.

A 62-year-old male with known peripheral arterial disease had undergone previous right to left femorofemoral bypass for claudication with a ringed PTFE graft (Figure 1) as well as subsequent thrombectomy of the fem-fem bypass, balloon angioplasty of the distal anastomosis, and stenting of the superficial femoral artery in March 2017 due to occlusion of the graft. Thirteen months later, the patient was presented with two-day history of fever, malaise, purulent drainage from previous thrombectomy incision, and new rest pain of the left lower extremity. Admission bloodwork did not demonstrate a leukocytosis or left shift. CT abdomen and pelvis demonstrated fluid surrounding the femorofemoral bypass graft (Figure 2). The bypass graft was occluded likely secondary to graft infection. Blood cultures demonstrated gram-positive bacteremia with associated fevers, and IV vancomycin was started. Transesophageal echocardiogram was performed which ruled out endocarditis. Due to previous graft thrombosis a year prior, the patient had been placed on oral anticoagulation which was held in preparation for surgery. The patient was taken to the operating room for left lower extremity revascularization and explantation of the infected femorofemoral bypass PTFE graft. Via a retroperitoneal approach, a left common iliac artery to above knee popliteal transobturator bypass was created with a 6 mm bovine carotid graft. The patient's postoperative course was uneventful. Preoperative rest pain was resolved with biphasic doppler signal distally of the posterior tibial and dorsalis pedis. Due to some difficulty with mobility, the patient was discharged to a rehabilitation facility. The patient was prescribed antiplatelet medication and a 4-week course of IV antibiotics via PICC for the staph aureus bacteremia. The patient was followed up outpatient following discharge with a repeat CT angiography of abdomen with bilateral lower extremity runoff at 2 months that demonstrated a widely patent transobturator bypass without evidence of stenosis or occlusion (Figures 3 and 4).

## 3. Discussion

Due to higher rates of amputation following ligation and explantation of infected bypass, revascularization should be the first priority after source control. Obturator bypass continues to be a viable and essential option when managing an infected groin. By avoiding the infected groin after explantation and performing revascularization in an uninfected field, the obturator bypass like other extra-anatomic options minimizes the risk of further infection. The obturator bypass however has an advantage over these other options such as the axillofemoral or iliolateral femoral bypasses with a superior secondary patency rate of 75% and 55% at 1 and 5 years, respectively [3].

Previous studies have demonstrated comparable patency rates at 2 years comparing saphenous vein graft and PTFE; however, on further follow-up at 5 years, greater saphenous vein grafts were the superior conduit type [4]. When choosing a conduit for obturator bypass, even though studies have shown that while autologous vein may serve as the best graft, this is not an option in every patient. Lindsey et al. found autologous vein was unsuitable for bypass in 72% of patients [5]. PTFE, however, is not the only alternative. An analysis of 124 lower extremity bypasses performed with bovine carotid artery graft whose proximal inflow vessels included external iliac, internal iliac, or common femoral, and target outflow vessels were above or below the knee popliteal or tibial arteries. This study determined that compared to autologous vein conduits, bovine carotid artery grafts demonstrated a comparable 5-year patency rate at 81% versus 68-83% [6].

Given the presence of bacteremia, synthetic conduit was not ideal for reconstruction. Our patient did have suitable saphenous vein; however, we chose to preserve this for future bypass due to the patient's progressive tibial disease demonstrated in Figure 3. Cryopreserved arterial allograft could have been considered; however, the ready availability of bovine carotid artery graft combined with its superior shelf life of three years and cost supported our choice in conduit [7, 8]. Also, the well-established use of bovine pericardial patch in infected fields with similar graft processing and storage in comparison to the bovine carotid artery further supported the use of the bovine carotid artery in the setting of infection [9, 10]. With comparable results to vein grafts in infrainguinal reconstructions, bovine carotid artery graft as demonstrated in our case should be considered a viable conduit choice for obturator bypass. We present our case as a potential index case for future investigation for uses of biogenic grafts.
